# Effect of microphysics scheme and data assimilation on hydrometeor and radiative flux simulations in the Arctic

**DOI:** 10.1098/rsos.240594

**Published:** 2024-09-04

**Authors:** Dae-Hui Kim, Hyun Mee Kim

**Affiliations:** ^1^ Atmospheric Predictability and Data Assimilation Laboratory, Department of Atmospheric Sciences, Yonsei University, Seoul, South Korea

**Keywords:** microphysics scheme, data assimilation, hydrometeor, radiative flux, arctic, cloud

## Abstract

Although clouds are a major factor influencing atmospheric environments in the Arctic, numerical simulations of Arctic clouds are uncertain. In this study, the effects of microphysics scheme and data assimilation (DA) on the simulation of clouds, hydrometeors and radiative fluxes in the Arctic were investigated using the polar weather research and forecasting (WRF) model and three-dimensional variational DA. Compared with the WRF 5-class (WSM5) microphysics scheme, when the Morrison double-moment (Morrison) scheme was used, the simulated amount of cloud ice water decreased by approximately 68%. In contrast, the amount of water vapour, cloud liquid water, snow and rain in the atmosphere increased. With DA, the amount of water vapour increased, leading to increased hydrometeors. The cloud liquid water increased in the middle and low atmospheres when Morrison was used, whereas it increased in the low atmosphere when DA was used. The increase in cloud liquid water by using Morrison resulted in a decrease in the downward short-wave radiative flux at the surface, whereas using DA increased the downward long-wave radiative flux. Changing the microphysics scheme induced redistribution of the region and amounts of hydrometeors, whereas DA induced an increase in hydrometeors in specific regions by adding observation information to the model states.

## 1. Introduction

Global warming is progressing more rapidly in the Arctic than in other regions [1,2]. Amplified warming in the Arctic is primarily caused by melted sea ice and decreased surface albedo in the Arctic [1] and modification of the atmospheric lapse rate [3]. Arctic warming and consequent changes in surface energy budgets [4] increase the amount of atmospheric water vapour [5,6], alter cloud properties [7–11], alter the thickness and stability of the planetary boundary layer (PBL) [12,13] and affect the intensity and pattern of the polar jet stream [14]. Fluctuations in the polar jet stream can cause extreme weather in mid-latitude regions [15,16]. Since the Arctic atmospheric environment has significant effects on various meteorological variables and phenomena on the globe, the Arctic atmospheric environment should be accurately simulated in numerical weather prediction (NWP) models.

Nevertheless, forecasts of the Arctic atmospheric environment are uncertain in NWP and climate models [17,18] partly due to the uncertain cloud simulations in numerical models [19,20]. Clouds affect downward radiative flux and surface energy budgets [21–23], and cloud radiative forcing depends on cloud composition [24]. In polar regions, when cloud ice water and supercooled cloud liquid water exist simultaneously, a realistic simulation of the cloud liquid water including supercooled cloud liquid water is important to determine the surface radiative flux [25,26]. Thus, a realistic simulation of the amount and distribution of cloud liquid water and cloud ice water in NWP models is necessary to accurately simulate Arctic atmospheric environments.

Previous studies investigated the characteristics of clouds simulated in polar regions in NWP models [27–34]. Microphysics schemes mainly affect the characteristics of the simulated clouds in NWP models. In NWP models, the use of the weather research and forecasting (WRF) single-moment five-class cloud microphysics scheme (WSM5), the WRF single-moment six-class cloud microphysics scheme (WSM6) and the WRF double-moment six-class cloud microphysics scheme (WDM6) as microphysics schemes resulted in underestimation of simulated cloud liquid water and overestimation of simulated cloud ice water [31–34]. When the number of ice nucleating particles (INPs) was large, more cloud ice water was simulated; thus, less supercooled cloud liquid water could be simulated in NWP models [35,36]. The influence of INPs on simulations of cloud ice water and cloud liquid water was shown for Antarctic cloud simulations using WSM5 in the polar weather research and forecasting (PWRF) model [31]. Listowski and Lachlan-Cope [31] also demonstrated that cloud ice water was overestimated using the single-moment microphysics scheme, which independently predicts only the mixing ratio of hydrometeors. However, the overestimation of cloud ice water decreased using the double-moment microphysics scheme, which independently predicts both the mixing ratio and number concentration of hydrometeors.

To reduce uncertainties in cloud simulations in polar regions, studies have been conducted to improve the microphysics schemes in NWP models [35,37–41]. Liu *et al*. [42] and Xie *et al*. [39] reduced cloud ice water and increased cloud liquid water by reducing INPs in the NCAR Community Atmospheric Model version 3 (CAM3) and NCAR Community Atmospheric Model version 5.1 (CAM5.1), respectively, and showed that the resulting cloud simulation was improved compared with observations. A single-moment microphysics scheme was used when the PWRF model was used as the operational NWP system (i.e. the Antarctic Mesoscale Prediction System; AMPS). However, the double-moment microphysics scheme was used in recent studies using the PWRF model [32,36]. To reduce the overestimation of cloud liquid water in the PWRF model, Hines and Bromwich [40] decreased the liquid cloud droplet concentration in a double-moment microphysics scheme based on the observed cloud condensation nuclei (CCN) concentration.

Compared with studies of microphysics schemes in polar cloud simulations, relatively few studies have been conducted using data assimilation (DA) to improve cloud simulations in the polar region [43]. Hines *et al*. [36] showed that the amount of water vapour increased in the initial condition; thus, the forecast errors of cloud liquid water and cloud ice water were reduced after nudging the rawinsonde observations of the upper atmosphere (i.e. horizontal wind speed, temperature and humidity) when simulating the atmospheric environment in Antarctica in the PWRF model. By performing satellite DA around Svalbard in the Arctic using the PWRF model with three-dimensional variational (3DVAR) DA, Kim and Kim [43] showed that the amount and distribution of water vapour became realistic in the initial condition, and thus, more cloud liquid water was predicted over a larger area around Svalbard. Studies without DA have also shown that more cloud liquid water can be simulated when the amount of water vapour in the atmosphere increases [30,34].

Although Kim and Kim [43] analysed clouds and hydrometeors to a limited extent in verifying the PWRF model results, Kim and Kim [43] focused on the verification of the radiative and heat fluxes at the surface simulated in the PWRF model based on observations. Therefore, no detailed analysis has been conducted to investigate the relationship between microphysical processes and DA in NWP models for simulating clouds and hydrometeors over the Arctic. In the forecasting process of NWP models, parts of the limited water vapour in the model atmosphere are changed to hydrometeors and precipitation, and the hydrometeors and precipitation are simulated differently depending on the microphysics schemes used in the NWP models [44]. DA causes a net inflow or outflow of water vapour in the simulated atmosphere by modifying the initial conditions of temperature and humidity using real observations. Thus, DA changes the amount and distribution of water vapour at the initial time in the model atmosphere, which changes the hydrometeor and precipitation forecasts [43]. Therefore, it is necessary to understand the characteristics of Arctic cloud simulations based on different microphysics schemes and DA. This understanding would be useful in determining the direction of research to supplement the limitations of microphysics schemes by developing microphysics schemes suitable for Arctic environments, by performing DA, or by doing together.

In this study, the effects of microphysics schemes and DA on the simulation of clouds and hydrometeors around Svalbard in the Arctic were investigated. The 3DVAR was used to perform DA, and two different microphysics schemes (i.e. WSM5 [45] and Morrison double-moment [46]) were used to generate forecasts in the PWRF model. The simulation results for clouds and hydrometeors in the PWRF model based on the two different microphysics schemes and DA are presented and discussed. Section 2 presents the methodology, and §3 presents the experimental results. Section 4 presents the summary and discussion.

## 2. Methodology

### 2.1. Model and data assimilation

In this study, the PWRF v. 3.7.1 model [27,28,47] was used. The PWRF model was developed to simulate polar atmospheric environments with a focus on two aspects. One is developing microphysics schemes to reduce the errors in forecasting polar clouds, and the other is developing surface processes with sea ice and snow information. The performance of the PWRF model was verified in polar regions [27,32,43,48,49], and the PWRF model is currently used in the AMPS and Arctic System Reanalysis v. 2 (ASRv2) production.

**Figure 1.**
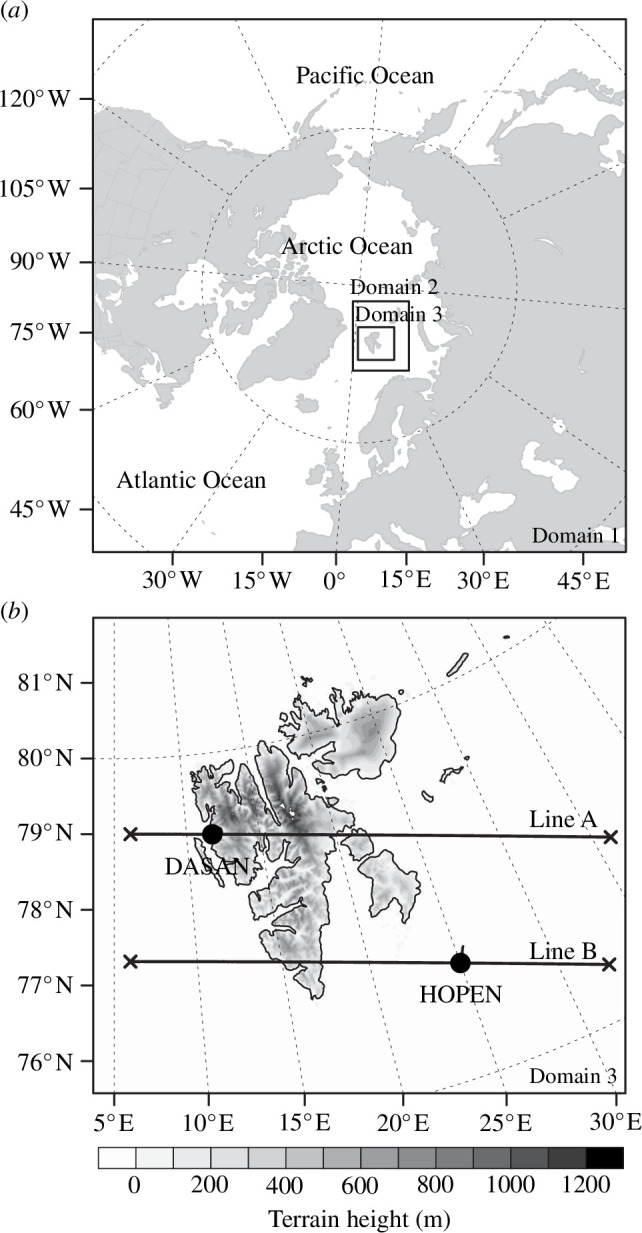
Experimental domain in this study: (*a*) domains 1, 2 and 3 and (*b*) domain 3 magnified. Vertical cross-sections along lines A and B were analysed. Lines A and B pass through DASAN station (78.9219 °N, 11.8658 °E) of the Republic of Korea in Ny-Ålesund and HOPEN station (76.5097 °N, 25.0133 °E), respectively. The locations of DASAN and HOPEN stations were marked with black circles.

The experimental model domains are shown in figure 1. The horizontal resolution and grid points of domain 1 were 15 km and 721 × 721, respectively. Domains 2 and 3 cover the Svalbard region of Norway with horizontal resolutions (grid points) of 5 km (283 × 229) and 1.67 km (400 × 442), respectively. All experimental results were analysed for domain 3. One-way nesting was applied between domains 1 and 2 and between domains 2 and 3. In all domains, the number of model vertical layers was 51 and the upper limit of the model was 10 hPa.

For all experiments in domain 1, the European Centre for Medium-Range Weather Forecasts (ECMWF) fifth-generation reanalysis (ERA5) [50] with a horizontal resolution of 0.25° × 0.25° was used as the initial and boundary conditions every 6 h (i.e. 00.00, 06.00, 12.00 and 18.00 UTC) during model integration. For all experiments in domain 2, the boundary conditions were calculated at each analysis time (i.e. 00.00, 06.00, 12.00 and 18.00 UTC) based on the one-way nesting. For the experiment with DA in domain 2, one-way nested values were used once at the beginning time of the model integration as the initial condition, and the following initial conditions were generated in analysis-forecast cycling using 3DVAR. In contrast, one-way nested values were used as the initial condition during the entire experimental period for the experiment without DA in domain 2. For all experiments in domain 3, the initial and boundary conditions were produced by the one-way nesting from domain 2. For all experiments, the sea surface temperature and sea ice cover were updated using ERA5 every 6 and 24 h, respectively. The default values for sea ice albedo and sea ice thickness in PWRF were 0.65 and 3 m, respectively.

The physics schemes were set appropriately for simulating the Arctic environments following Bromwich *et al*. [51] and Kim and Kim [43] and were the same for all experimental domains except for the cumulus parametrization scheme. The Noah land surface model [52] optimized for the polar region was used for land surface parametrization, Monin and Obukhov [53] was used for surface layer parametrization, Mellor–Yamada–Janjic turbulent kinetic energy [54] was used for PBL parametrization, and the rapid radiative transfer model for global climate models [55] was used for short-wave and long-wave radiation parametrization. For cumulus parametrization, the Grell–Dévényi ensemble [56] was used only for domain 1 and not for domains 2 and 3. In the NWP model, a 5 km horizontal resolution is considered ‘the lower end of the grey zone of convection’ [57]. Some studies have been conducted using a 5 km horizontal resolution in NWP models without utilizing the cumulus scheme [31,57]. To analyse and compare the effects of different microphysics schemes on simulating hydrometeors in PWRF, WSM5 as a single-moment microphysics scheme and Morrison double-moment (hereafter Morrison) as a double-moment microphysics scheme optimized for the polar region were used. WSM5 has been used in PWRF for AMPS. Morrison has been widely used in PWRF, and the performance of the Morrison for cloud simulation in polar regions has been verified in several studies (e.g. [28,32,40].

Using the WRFDA v. 3.8 3DVAR system [58], DA was performed every 6 h in domain 2 due to the following reasons: domain 3 has a much higher horizontal resolution than the distance between satellite data used for DA; domain 2 has more available observational data for DA compared with domain 3; By performing DA in domain 2, the changes in atmospheric conditions upstream of domain 3 can be considered in the forecasts for domain 3. Observations within ±3 h at each analysis time were used for DA. For satellite DA, the community radiative transfer model (CRTM) was used as the radiative transfer model, and variational bias correction was applied. The background error covariance for 3DVAR DA was calculated based on the National Meteorological Center (NMC) method [59], using the differences between the 12 and 24 h forecasts for September 2017.

### 2.2. Observation data

Conventional observations (i.e. radiosonde, surface synoptic observation from land station including aviation weather report, surface synoptic observation from ship and buoy, atmospheric motion vector and scatterometer sea surface wind) and microwave radiance data (Advanced Microwave Sounding Unit-A (ASMU-A) and microwave humidity sounder (MHS)) from satellites that are assimilated to the National Centers for Environmental Prediction (NCEP) Global Data Assimilation System (GDAS) were used for DA. The AMSU-A and MHS are sensitive to the vertical temperature and humidity distributions, respectively. The satellite observation channels used for DA were the AMSU-A channels 5–9 and MHS channels 3–5, which were used for ASRv2 production. Considering the quality of the satellite observation channels (https://www.emc.ncep.noaa.gov/mmb/data_processing/Satellite_Historical_Documentation.htm), AMSU-A channels 7 and 8 in METOP-2, channel 9 in NOAA-18, channel 8 in NOAA-19, and MHS channel 3 in NOAA-19 were not used for DA. The thinning distances of the AMSU-A and MHS were 90 and 60 km, respectively.

The cloud liquid water and ice water contents included in Radar-Only Cloud Water Content Product level 2B (2B-CWC-RO) [60], which are products from CloudSat, were used to evaluate the vertical distributions of cloud liquid water and ice water contents simulated in each experiment.

### 2.3. Experimental framework

The experimental configuration is listed in table 1. For the microphysics scheme, EXP1 and EXP2 used WSM5, and EXP3 and EXP4 used Morrison. Both EXP1 and EXP3 were performed in the absence of DA. In EXP1 and EXP3, 30 h forecasts were produced at every analysis time (i.e. 00.00, 06.00, 12.00 and 18.00 UTC) in domain 2 using ERA5 as the initial and boundary conditions for PWRF. DA was performed in both EXP2 and EXP4. In EXP2 and EXP4, the analysis was produced every 6 h (i.e. 00.00, 06.00, 12.00 and 18.00 UTC) in domain 2 using 3DVAR, and the 30 h forecasts were produced every analysis time (i.e. 00.00, 06.00, 12.00 and 18.00 UTC) using the analysis produced in 3DVAR as the initial condition and ERA5 as the boundary condition for PWRF. In all the experiments, the forecasts for domain 3 were produced by applying one-way nesting to domain 2.

**Table 1.** Experimental configuration.

experiment	microphysics scheme	type	observation
EXP1	WSM5	Without DA	N/A
EXP2		With DA	conventional observation + microwave radiance (AMSU-A, MHS)
EXP3	Morrison double-moment	Without DA	N/A
EXP4		With DA	conventional observation + microwave radiance (AMSU-A, MHS)

The experimental results were analysed for domain 3. The analysis period was 6−19 September 2017. For experiments with DA (i.e. EXP2 and EXP4), own analysis-forecast cycling was started on 1 September 2017 to have 5 days of spin-up period. The first 24 h of the 30 h forecasts were not analysed because it takes approximately 24 h to develop the PBL reflecting the surface characteristics and to adapt the initial conditions to the hydrologic cycle in the Arctic in the PWRF [28,43,49]. Thus, the experimental results were analysed by 1 h interval time-series data from 25 to 30 h forecasts at 00.00, 06.00, 12.00 and 18.00 UTC. To investigate the averaged characteristics during the experimental period, 1 h interval time-series data were averaged for the experimental period from 6 to 19 September 2017.

To verify whether the experimental results were significantly different, a significance test was performed at 99% and 95% confidence levels using repeated measures analysis of variance (RM ANOVA) [61]. After calculating the RM ANOVA, a post hoc test using the Bonferroni method [62–65] was performed to verify which experimental results were significantly different among the four experiments. When conducting a significance test, as the sample size increases, it becomes easier to detect significant differences among multiple datasets. To perform significance tests at an appropriate sample size, time-series data averaged at 6 h intervals for each variable were used to conduct the significance test.

## 3. Results

### 3.1. Effect of microphysics scheme and data assimilation on hydrometeors

#### 3.1.1. Time series

**Figure 2.**
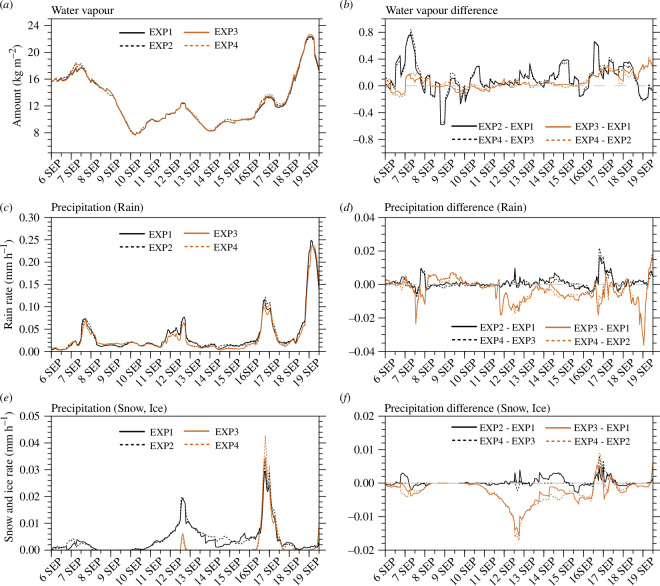
Time series of 25−30 h forecasts in EXP1, EXP2, EXP3 and EXP4 for (*a*) water vapour (kg m^−2^), (*c*) precipitation with rain (mm h^−1^) and (*e*) precipitation with snow and ice (mm h^−1^). Time series of differences in 25−30 h forecasts between experiments for (*b*) water vapour (kg m^−2^), (*d*) precipitation with rain (mm h^−1^) and (*f*) precipitation with snow and ice (mm h^−1^). The water vapour and precipitations were averaged every hour over the domain 3.

**Figure 3.**
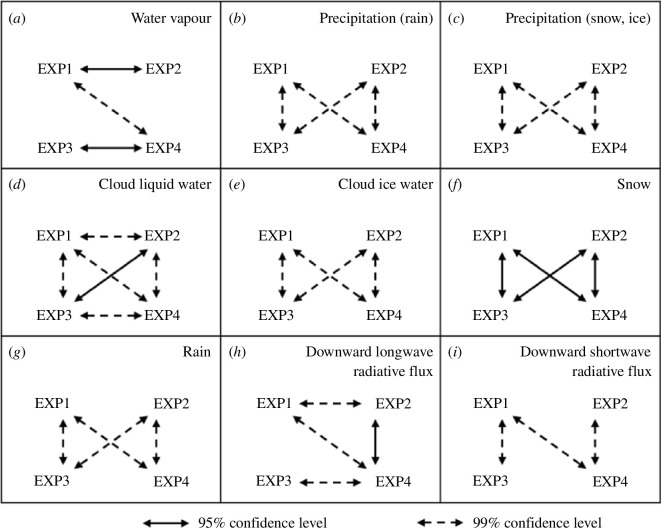
Summary of significant tests for time series data: (*a*) water vapour in *figure 2a,* (*b*) precipitation (rain) in *figure 2b,* (*c*) precipitation (snow and ice) in *figure 2c,* (*d*) cloud liquid water in figure 4*a,* (*e*) cloud ice water in *figure 4b,* (*f*) snow in figure 4*c,* (*g*) rain in *figure 4d,* (*h*) downward long-wave radiative flux in *figure 11a* and (*i*) downward short-wave radiative flux in *figure 11b*. The time series data averaged at 6 h intervals were used for significance tests.

Figure 2 shows the time series of the averaged amount of water vapour and precipitation simulated in domain 3 at 1 h intervals for each experiment, along with the differences between the experiments. The water vapour amount averaged during the experimental period was 12.60, 12.71, 12.66 and 12.78 kg m^−2^ in EXP1, EXP2, EXP3 and EXP4, respectively. The amount of water vapour was significantly different at the 95% confidence level between EXP1 and EXP2 and between EXP3 and EXP4, which implies that the total amount of water vapour simulated in domain 3 increased significantly when DA was performed (figure 3*a*). In addition, the amount of water vapour was significantly different at the 99% confidence level between EXP1 and EXP4, which implies that the total amount of water vapour increased significantly when DA was performed with Morrison (figure 3*a*).

**Figure 4.**
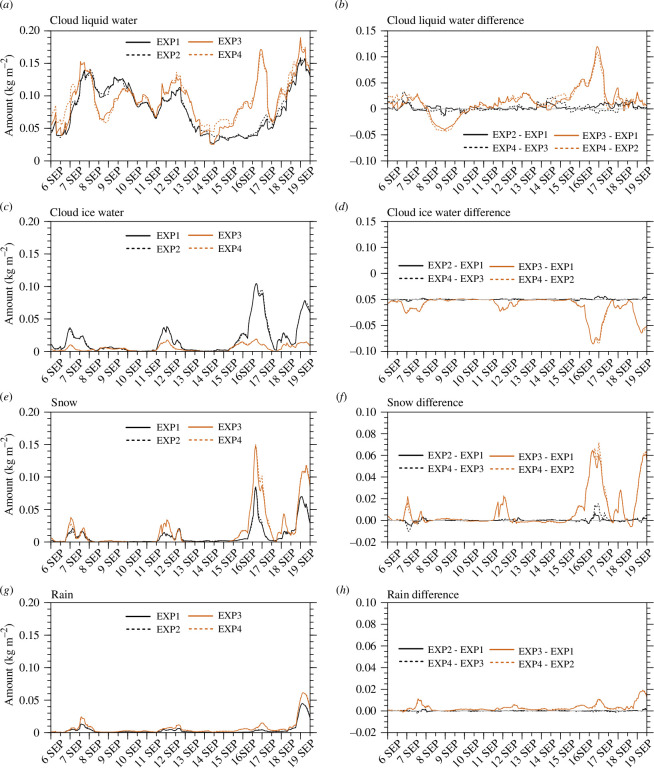
Time series of 25−30 h forecasts in EXP1, EXP2, EXP3 and EXP4 for (*a*) cloud liquid water (kg m^−2^), (*c*) cloud ice water (kg m^−2^), (*e*) snow (kg m^−2^) and (*g*) rain (kg m^−2^). Time series of differences in 25−30 h forecasts between experiments for (*b*) cloud liquid water (kg m^−2^), (*d*) cloud ice water (kg m^−2^), (*f*) snow (kg m^−2^) and (*h*) rain (kg m^−2^). Cloud liquid water, cloud ice water, snow and rain were averaged every hour over the domain 3.

When the microphysics scheme was changed from WSM5 to Morrison, the amount of water vapour increased by +0.48% (+0.55%) in EXP3 (EXP4) compared with EXP1 (EXP2). Compared with EXP1 (EXP3), the amount of water vapour increased by +0.87% (+0.95%) in EXP2 (EXP4) when DA was performed. Therefore, water vapour increased more when DA was performed than when Morrison was used instead of WSM5. During the experimental period, the magnitude of the water vapour fluctuation was greater when DA was performed compared with the case when microphysics scheme was changed (figure 2*b*).

The precipitation rates for rain at the surface averaged over the domain 3 during the experimental period were 34.84, 35.96, 31.25 and 32.23 (×10^−3^) mm h^−1^ in EXP1, EXP2, EXP3 and EXP4, respectively (figure 2*c*). The precipitation rates for snow and ice at the surface averaged over the domain 3 during the experimental period were 3.89, 4.17, 1.44 and 1.66 (×10^−3^) mm h^−1^ in EXP1, EXP2, EXP3 and EXP4, respectively (figure 2*e*). The precipitation rates for rain, snow and ice at the surface in each experiment were significantly different at the 99% confidence level between experiments with different microphysics schemes (figure 3*b,c*), which implies that the total precipitation rates decreased significantly when the microphysics scheme was changed from WSM5 to Morrison. In summer, precipitation in Svalbard is mostly simulated as rain, rather than snow and ice. When the microphysics scheme was changed from WSM5 to Morrison, the precipitation rate for rain and those for snow and ice decreased by −10.30% and −62.98% (−10.37% and −60.19%) in EXP3 (EXP4) compared with those in EXP1 (EXP2), respectively. When DA was performed, the precipitation rates for rain and those for snow and ice increased by +3.21% and +7.20% (+3.14% and +15.28%) in EXP2 (EXP4) compared with those in EXP1 (EXP3), respectively. In the experiments using Morrison, almost no precipitation rates for snow and ice were simulated, except on 12 and 16−17 September (figure 2*e*). As the microphysics scheme changed from WSM5 to Morrison, the decreasing features of precipitation for rain, snow and ice continued throughout the experimental period regardless of the DA (figure 2*d,f*). Since limited amounts of atmospheric water vapour change to atmospheric hydrometeors and precipitation during the forecasting process in the NWP model, less precipitation at the surface when using Morrison instead of WSM5 may result in more atmospheric hydrometeors simulated. When DA was performed, increasing features of precipitation for rain, snow and ice appeared throughout the experimental period, regardless of the microphysics schemes (figure 2*d,f*).

Figure 4 shows the time series of the average amount of cloud liquid water, cloud ice water, snow and rain simulated in domain 3 at 1 h intervals for each experiment, along with the differences between the experiments. During the experimental period, the average amount of cloud liquid water simulated was 79.28, 81.68, 92.93 and 96.01 (×10^−3^) kg m^−2^ (figure 4*a*), cloud ice water 17.61, 17.84, 4.98 and 5.00 (×10^−3^) kg m^−2^ (figure 4*c*), snow 9.12, 9.17, 17.03 and 17.36 (×10^−3^) kg m^−2^ (figure 4*e*), rain 3.76, 3.83, 6.74 and 6.79 (×10^−3^) kg m^−2^ (figure 4*g*) in EXP1, EXP2, EXP3 and EXP4, respectively. The average values of each hydrometeor were significantly different in each experiment at the 99% confidence level for all experiments.

The average values of cloud liquid water, cloud ice water, snow and rain were significantly different at above 95% confidence level between experiments with different microphysics scheme (figure 3*d*–*g*). Only the average values of cloud liquid water were significantly different at the 99% confidence level between experiments without and with DA (i.e. between EXP1 and EXP2 and between EXP3 and EXP4) (figure 3*d*). Around Svalbard in summer, cloud liquid water was simulated more than cloud ice water.

When the microphysics scheme was changed from WSM5 to Morrison, cloud liquid water, snow and rain increased by +17.22%, +86.73% and +79.26% (+17.54%, +89.31% and +77.28%) in EXP3 (EXP4) compared with EXP1 (EXP2), respectively, and cloud ice water decreased by −71.72% (−71.97%) in EXP3 (EXP4) compared with EXP1 (EXP2). Thus, when Morrison was used instead of WSM5, cloud liquid water, snow and rain increased and cloud ice water decreased. The number concentration of INPs is overestimated in an INP parametrization scheme used for the deposition process when using WSM5 in the NWP model, which results in the limited water vapour in the atmosphere being simulated mostly as cloud ice water [31]. In the forecasting process using WSM5, the overestimation of cloud ice water was maintained because the number concentration of cloud ice water at the next time step was proportional to the mixing ratio of cloud ice water at the previous time step on a logarithmic scale. In Morrison, the number concentration of INPs is not diagnosed excessively in an INP parametrization scheme, and the number concentration of cloud ice water is predicted independently during the forecasting process. Therefore, when Morrison was used instead of WSM5, less cloud ice water and more cloud liquid water, snow and rain were simulated. Exceptionally, less cloud liquid water was simulated in EXP3 and EXP4 than in EXP1 and EXP2 during 8−9 September (figure 4*a,b*). This is because precipitation for rain was simulated more in EXP3 and EXP4 than in EXP1 and EXP2 at that time (figure 2*d*). The microphysics scheme distributes the limited atmospheric water vapour to hydrometeors and precipitation, and the amounts of simulated hydrometeors and precipitation were significantly different depending on the microphysics scheme (figure 3*b–g*).

When DA was performed, cloud liquid water, cloud ice water, snow and rain increased by +3.03%, +1.31%, +0.55% and +1.86% (+3.31%, +0.40%, +1.94% and +0.74%) in EXP2 (EXP4) compared with EXP1 (EXP3), respectively. After DA, the amounts of hydrometeors increased, regardless of the microphysics schemes used. The increase in the water vapour amounts at the initial time of model integration by assimilating the observation data led to an increase in the hydrometeor amounts at the forecast time in the NWP model.

#### 3.1.2. Horizontal distribution

**Figure 5.**
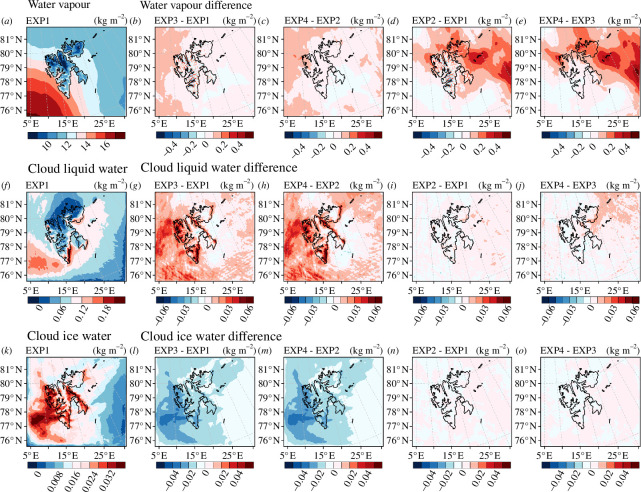
Vertically integrated water vapour (kg m^−2^) for domain 3: (*a*) EXP1, (*b*) difference between EXP3 and EXP1, (*c*) difference between EXP4 and EXP2, (*d*) difference between EXP2 and EXP1 and (*e*) difference between EXP4 and EXP3. Vertically integrated cloud liquid water (kg m^−2^) for domain 3: (*f*) EXP1, (*g*) difference between EXP3 and EXP1, (*h*) difference between EXP4 and EXP2, (*i*) difference between EXP2 and EXP1 and (*j*) difference between EXP4 and EXP3. Vertically integrated cloud ice water (kg m^−2^) for domain 3: (*k*) EXP1, (*l*) difference between EXP3 and EXP1, (*m*) difference between EXP4 and EXP2, (*n*) difference between EXP2 and EXP1 and (*o*) difference between EXP4 and EXP3. The vertically integrated water vapour and hydrometeors were averaged for the experimental period of 6−19 September.

Figure 5 shows the horizontal distributions of the vertically integrated water vapour, cloud liquid water and cloud ice water in domain 3 averaged during the experimental period in each experiment and the differences between the experiments. The simulated water vapour was the largest in the southwest of Svalbard and decreased towards the northeast (figure 5*a*). The cloud liquid water was simulated more in the east than in the west of Svalbard (figure 5*f*) due to the easterly winds during the experimental period. Cloud ice water was simulated more over Svalbard than over the sea (figure 5*k*).

The water vapour and cloud liquid water increased when Morrison was used instead of WSM5 (figure 5*b,c*,*g*,*h*) and when DA was performed (figure 5*d,e*,*i,j*). Water vapour increased more when DA was performed than the case when Morrison was used (figure 5*b–e*), whereas cloud liquid water increased more when Morrison was used than when DA was performed (figure 5*g–j*). Cloud ice water decreased remarkably when Morrison was used instead of WSM5 (figure 5*l,m*). However, cloud ice water increased very slightly when DA was performed; thus, the differences in simulated cloud ice water between the experiments were not evident (figure 5*n,o*). When Morrison was used instead of WSM5, cloud ice water decreased, cloud liquid water increased (figure 5*g,h*,*l,**m*) and water vapour increased in the area where cloud ice water decreased (figure 5*b,c*,*l,**m*). The water vapour increased north and east of Svalbard when DA was performed (figure 5*d,e*). The area where the water vapour increased by performing DA was not related to the area where the amount of water vapour changed as the microphysics scheme was changed (figure 5*b–e*). The DA modified the amount and distribution of atmospheric water vapour in the initial conditions of the NWP model by assimilating observational data, which is independent of microphysics schemes. When DA was performed, cloud liquid water tended to increase in areas where water vapour increased (figure 5*d,e*,*i,**j*).

#### 3.1.3. Vertical distribution

Two vertical cross-sections were drawn along lines A and B to investigate the vertical distributions of hydrometeors in each experiment (figure 1). Lines A and B pass the DASAN station (78.9219° N and 11.8658° E) of the Republic of Korea at Ny-Ålesund and the HOPEN station (76.5097° N and 25.0133° E), respectively.

**Figure 6.**
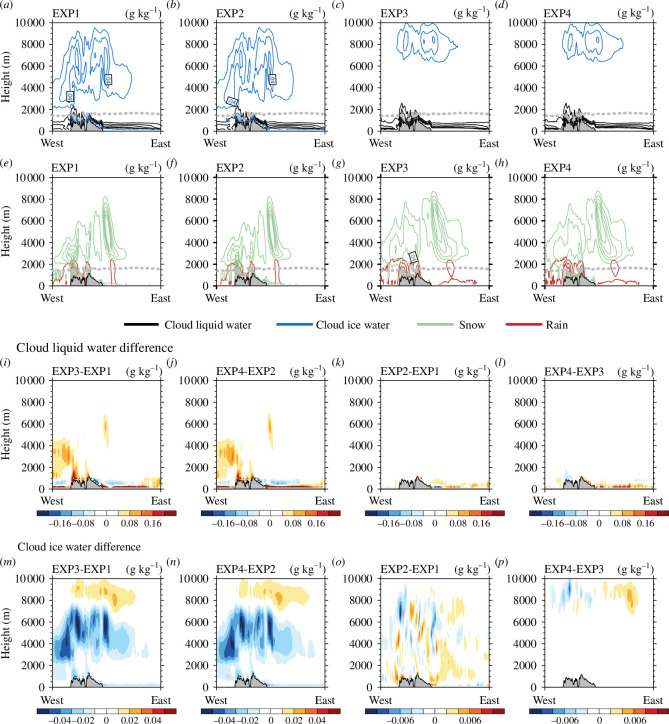
Vertical cross-sections of cloud liquid water (g kg^−1^) and cloud ice water (g kg^−1^) along line A in (*a*) EXP1, (*b*) EXP2, (*c*) EXP3 and (*d*) EXP4. Vertical cross-sections of snow (g kg^−1^) and rain (g kg^−1^) along line A in (*e*) EXP1, (*f*) EXP2, (*g*) EXP3 and (*h*) EXP4. The freezing level is indicated by a grey dashed line. The differences of vertical cross-sections for cloud liquid water (g kg^−1^): (*i*) difference between EXP3 and EXP1, (*j*) difference between EXP4 and EXP2, (*k*) difference between EXP2 and EXP1 and (*l*) difference between EXP4 and EXP3. The differences of vertical cross-sections for cloud ice water (g kg^−1^): (*m*) difference between EXP3 and EXP1, (*n*) difference between EXP4 and EXP2, (*o*) difference between EXP2 and EXP1 and (*p*) difference between EXP4 and EXP3. The vertical cross-sections of hydrometeors and freezing levels were averaged for the experimental period of 6−19 September.

Figure 6 shows the vertical cross-sections of hydrometeors simulated in each experiment along line A (figure 1) averaged during the experimental period and the differences between the experiments. Along line A, the average amount of cloud liquid water path simulated was 62.34, 65.78, 72.74 and 75.54 g m^−2^ and cloud ice water path 15.44, 15.75, 5.62 and 5.69 g m^−2^ in EXP1, EXP2, EXP3 and EXP4, respectively (figure 6*a–d*). Along line A, when Morrison was used instead of WSM5, cloud liquid water increased by +16.68% (+14.84%) and cloud ice water decreased by −63.60% (−63.87%) in EXP3 (EXP4) compared with EXP1 (EXP2). When DA was performed, cloud liquid water increased by +5.52% (+3.85%) and cloud ice water increased by +2.01% (+1.25%) in EXP2 (EXP4) compared with EXP1 (EXP3). Along line A, cloud liquid water and rain were mainly simulated below 2000 m, whereas cloud ice water and snow were mainly simulated above 2000 m (figure 6*a–h*). Cloud liquid water was simulated over a large area in the lower atmosphere up to approximately 1000 m above sea and land (figure 6*a–d*). When Morrison was used instead of WSM5, cloud ice water decreased around 2000−6000 m and snow increased in these layers (figure 6*c,d*,*g,**h*). The threshold value of the size at which cloud ice water changes to snow is smaller in Morrison than in WSM5, thus, less cloud ice water and more snow were simulated in Morrison than in WSM5 [31]. Figure 6*i–p* shows the differences in cloud liquid water and cloud ice water between the experiments in the vertical cross-sections during the experimental period. When Morrison was used instead of WSM5, cloud liquid water increased in the lower atmosphere and west of Svalbard, where cloud ice water decreased (figure 6*i,j*), and cloud ice water decreased in most vertical layers (figure 6*m,n*). When DA was performed, cloud liquid water increased in the lower atmosphere over the eastern sea of Svalbard (figure 6*k,l*), and cloud ice water changed broadly in the atmosphere (figure 6*o*) with WSM5 and in the upper atmosphere with Morrison (figure 6*p*). Note that the differences caused by performing DA were approximately an order of magnitude smaller than those caused by changing the microphysics scheme.

**Figure 7.**
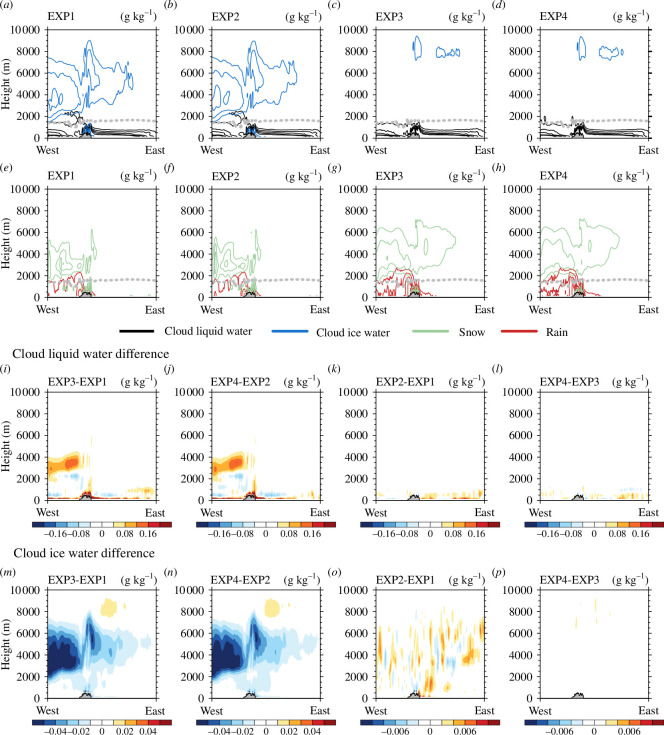
Vertical cross-sections of cloud liquid water (g kg^−1^) and cloud ice water (g kg^−1^) along line B in (*a*) EXP1, (*b*) EXP2, (*c*) EXP3 and (*d*) EXP4. Vertical cross-sections of snow (g kg^−1^) and rain (g kg^−1^) along line B in (*e*) EXP1, (*f*) EXP2, (*g*) EXP3 and (*h*) EXP4. The freezing level is indicated by a grey dashed line. The differences of vertical cross-sections for cloud liquid water (g kg^−1^): (*i*) difference between EXP3 and EXP1, (*j*) difference between EXP4 and EXP2, (*k*) difference between EXP2 and EXP1 and (*l*) difference between EXP4 and EXP3. The differences of vertical cross-sections for cloud ice water (g kg^−1^): (*m*) difference between EXP3 and EXP1, (*n*) difference between EXP4 and EXP2, (*o*) difference between EXP2 and EXP1 and (*p*) difference between EXP4 and EXP3. The vertical cross-sections of hydrometeors and freezing level were averaged for the experimental period of 6−19 September.

Figure 7 shows the vertical cross-sections of hydrometeors simulated in each experiment along line B (figure 1) averaged during the experimental period and the differences between the experiments. Along line B, the average amounts of cloud liquid water path simulated were 79.60, 82.79, 88.23 and 89.79 g m^−2^ and cloud ice water path 16.67, 17.32, 3.77 and 3.85 g m^−2^ in EXP1, EXP2, EXP3 and EXP4, respectively (figure 7*a–d*). Along line B, when Morrison was used instead of WSM5, cloud liquid water increased by +10.84% (+8.46%) and cloud ice water decreased by −77.38% (−77.78%) in EXP3 (EXP4) compared with EXP1 (EXP2). When DA was performed, cloud liquid water increased by +4.01% (+1.77%) and cloud ice water increased by +3.90% (+2.12%) in EXP2 (EXP4) compared with EXP1 (EXP3). The characteristics of the vertical distribution of hydrometeors according to the microphysics schemes used and the DA were similar along lines A and B (figures 6,7 and 7), indicating that these characteristics were not limited to specific cross-sections.

Similar to the time series and horizontal distribution, in the vertical cross-section, cloud liquid water, snow and rain increased, whereas cloud ice water decreased when Morrison was used instead of WSM5, and all hydrometeors increased when DA was performed. These two features appear consistently along lines A and B.

The vertical cross-sections of cloud liquid water and ice water contents simulated in EXP1, EXP2, EXP3 and EXP4 were evaluated using those observed in the CloudSat 2B-CWC-RO. Table 2 shows the observation times for CloudSat 2B-CWC-RO, which is valid on domain 3 from 06.00 UTC 16 September to 06.00 UTC 17 September 2017. Since the amounts of cloud liquid and ice water simulated in each experiment were very different for the period (figure 4*a,c*), the period is appropriate to analyse the effect of different microphysics schemes on cloud simulations.

**Table 2.** Times of CloudSat 2B-CWC-RO observations used to evaluate the cloud liquid water and ice water contents simulated in each experiment.

date	time (h.min)
16 September 2017	09.13 UTC
17 September 2017	01.42 UTC

**Figure 8.**
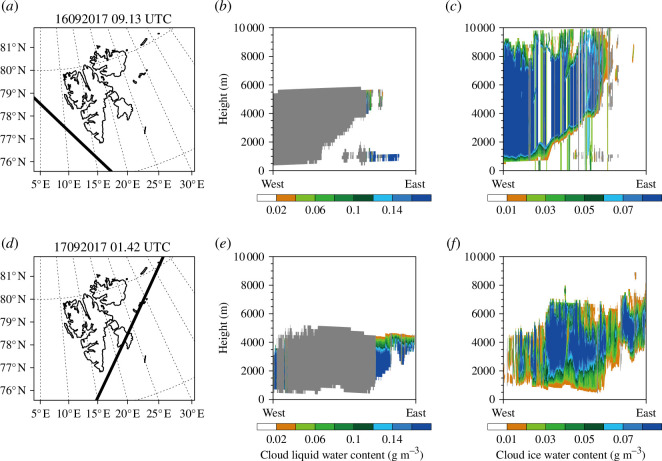
(*a*) CloudSat observation path at 09.13 UTC 16 September 2017. Vertical cross-sections of (*b*) cloud liquid water content (g m^−3^) and (*c*) cloud ice water content (g m^−3^) from the CloudSat observations at 09.13 UTC 16 September 2017. (*d–f*) The same as (*a–c*) but for observation time of 01.42 UTC 17 September 2017. The grey shading in (*b*) and (*e*) indicates the quality of the CloudSat observation is not good enough.

**Figure 9.**
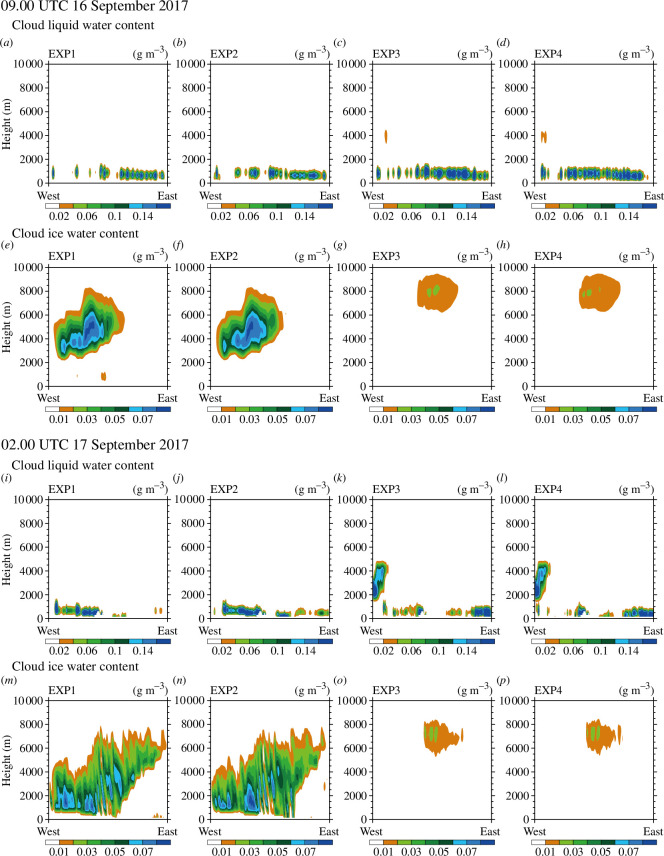
Vertical cross-sections of cloud liquid water content (g m^−3^) in (*a*) EXP1, (*b*) EXP2, (*c*) EXP3, (*d*) EXP4 and cloud ice water content (g m^−3^) in (*e*) EXP1, (*f*) EXP2, (*g*) EXP3, (*h*) EXP4 at 09.00 UTC 16 September 2017. (*i–p*) The same as (*a-h*) but for 02.00 UTC 17 September 2017.

Figure 8 shows the CloudSat observation path (figure 8*a,d*), the vertical cross-sections of cloud liquid water and ice water contents observed by CloudSat at each time in table 2. Figure 9 shows the vertical cross-sections of cloud liquid water and ice water contents simulated in EXP1, EXP2, EXP3 and EXP4 at the closest times (i.e. 09.00 UTC 16 September and 02.00 UTC September 2017) to each observation time shown in table 2.

The observed cloud liquid water was noticed at altitudes of approximately 500–6000 m (figure 8*b,e*), whereas the simulated cloud liquid water contents in all experiments were mainly shown below 2000 m (figure 9*a–d* and *i–l*). When using Morrison instead of WSM5, the cloud liquid water was simulated above 2000 m slightly (figure 9*c*,*d*,*k*,). The observed cloud ice water was shown above 1000 m (figure 8*c,f*). Although the cloud ice water contents were underestimated in all experiments (figure 9*e–h* and *m–p*), the simulated cloud ice water using WSM5 was more similar to observations than Morrison (figure 9*e,f*,*m,n*). In Morrison, cloud ice water was slightly simulated (figure 9*g,h*,*o,**p*). Overall, the cloud liquid water was simulated more similar to the CloudSat observations when using Morrison instead of WSM5, whereas cloud ice water was simulated more similar to the CloudSat observations when using WSM5 instead of Morrison (figures 8,9 and 9). The relative amount of cloud liquid and ice water simulated in experiments were consistent with the vertical cross-sections along lines A and B (figures 6,7 and 7). Unlike in Antarctica where WSM5 overestimated cloud ice water [31], WSM5 may not overestimate cloud ice water in the Arctic.

Regardless of the microphysics schemes, cloud liquid water was simulated more at altitudes approximately below 1000 m when DA was performed (figure 9*b,d,j*), whereas the amount of cloud ice water changed slightly. This is consistent with the significantly increased cloud liquid water when DA was performed, unlike slightly increased cloud ice water (figures 3,4 and 4).

#### 3.1.4. Vertical profiles averaged horizontally

**Figure 10.**
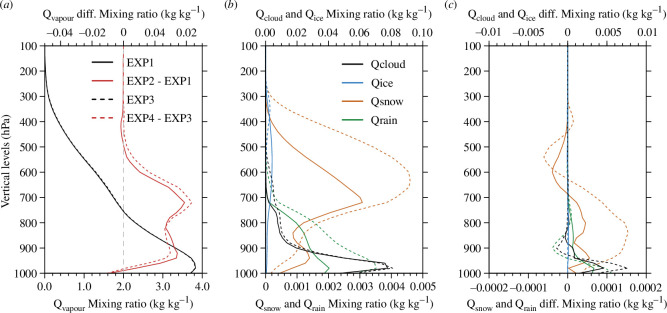
(*a*) The vertical distribution of horizontally averaged water vapour (Q_vapour_) (kg kg^−1^) for domain 3 in EXP1 and EXP3 and its differences in EXP2 (EXP4) against EXP1 (EXP3). (*b*) The vertical distributions of horizontally averaged hydrometeors (Q_cloud_, Q_ice_, Q_snow_ and Q_rain_) (kg kg^−1^) for domain 3 in EXP1 and EXP3. (*c*) The differences of vertical distributions for horizontally averaged hydrometeors (kg kg^−1^) in EXP2 (EXP4) against EXP1 (EXP3). The vertical distributions of horizontally averaged water vapour and hydrometeors were averaged for the experimental period of 6−19 September. For (*b*) and (*c*), EXP1 and EXP2 are denoted by solid lines, whereas EXP3 and EXP4 are denoted by dashed lines. Note that Q_cloud_, Q_ice_, Q_snow_ and Q_rain_ mean cloud liquid water, cloud ice water, snow and rain, respectively.

The effects of the microphysics scheme and DA on hydrometeor simulations were also investigated using the vertical profiles of the simulated hydrometeors in domain 3. Figure 10*a* shows the vertical profiles of the water vapour horizontally averaged in domain 3 and temporally averaged during the experimental period and the differences between the experiments. In EXP1 and EXP3, the amount of water vapour was the greatest below 900 hPa and decreased as the altitude increased (figure 10*a*). When DA was performed in EXP2 and EXP4, water vapour increased below 400 and 500 hPa compared with EXP1 and EXP3, respectively (figure 10*a*). Figure 10*b* shows the vertical profiles of the hydrometeors horizontally averaged in domain 3 and temporally averaged over the experimental period in EXP1 and EXP3. The cloud ice water in the 400−800 hPa layer decreased in EXP3 compared with that in EXP1. Compared with EXP1, cloud liquid water, snow and rain increased in most layers in EXP3, except for the decrease in snow in the layer below 900 hPa. Thus, when Morrison was used instead of WSM5, cloud ice water decreased and other hydrometeors, except cloud ice water, increased in domain 3. Figure 10*c* shows the differences in the hydrometeors simulated in EXP2 and EXP4 compared with EXP1 and EXP3, respectively. When DA was performed in EXP2 and EXP4, cloud liquid water, cloud ice water, snow and rain increased compared with EXP1 and EXP3, respectively (figure 10*c*). Therefore, the simulated hydrometeors increased when DA was performed in domain 3.

In the lower atmosphere below 900 hPa, cloud liquid water increased when Morrison was used instead of WSM5 (figure 10*b*) and increased when DA was performed (figure 10*c*). As the microphysics scheme changed from WSM5 to Morrison, the cloud liquid water increased similarly in the middle and lower atmosphere. However, when DA was performed, cloud liquid water increased intensively in the lower atmosphere below 900 hPa. Therefore, DA could be effective in simulating more cloud liquid water in the lower atmosphere over the Arctic region, where cloud liquid water was underestimated in most NWP models. Furthermore, when DA was performed with Morrison rather than with WSM5, more cloud liquid water was simulated in the lower atmosphere over the Arctic. Therefore, changing the microphysics scheme from WSM5 to Morrison induced redistribution of the region and amounts of hydrometeors. In contrast, DA induced an increase in cloud liquid water in specific regions by adding observation information to the model states.

### 3.2. Effect of hydrometeor variations by microphysics scheme and data assimilation on radiative fluxes

The amount and distribution of hydrometeors changed by microphysics schemes and DA can affect the downward radiative flux simulations in the Arctic. The effect of hydrometeors on the downward radiative flux is important to understand and reduce the uncertainties associated with atmospheric forecasts in the Arctic.

**Figure 11.**
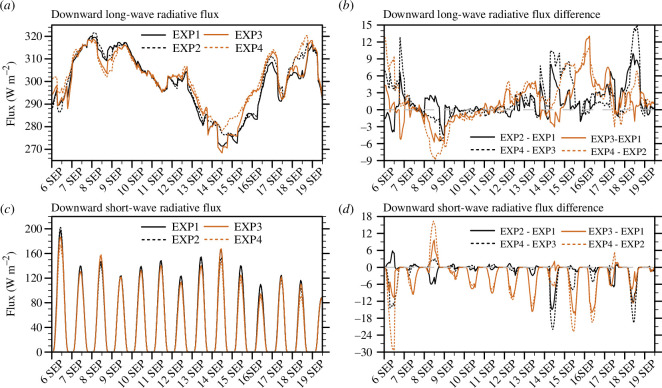
Time series of 25−30 h forecasts in EXP1, EXP2, EXP3 and EXP4 for (*a*) downward long-wave radiative flux (LWD; W m^−2^), and (*c*) downward short-wave radiative flux (SWD; W m^−2^). Time series of differences in 25−30 h forecasts between experiments for (*b*) LWD (W m^−2^), and (*d*) SWD (W m^−2^). The radiative fluxes were averaged every hour over the domain 3.

Figure 11 shows the time series of the average amount of downward radiative flux simulated in domain 3 at 1 h intervals for each experiment and the differences between the experiments. The average downward long-wave radiative fluxes (LWDs) simulated during the experimental period were 299.04, 300.32, 300.15 and 302.00 W m^−2^ in EXP1, EXP2, EXP3 and EXP4, respectively. The average downward short-wave radiative fluxes (SWDs) were 47.55, 46.66, 45.01 and 43.51 W m^−2^ in EXP1, EXP2, EXP3 and EXP4, respectively. The LWDs were significantly different at above 95% confidence level, except for those between EXP1 and EXP3, and EXP2 and EXP3 (figure 3*h*), implying that performing DA had a more significant effect than changing the microphysics scheme on the increase in LWDs. The SWDs were significantly different at the 99% confidence level between EXP1 and EXP3, EXP2 and EXP4 and EXP1 and EXP4 (figure 3*i*), implying that changing microphysics scheme had a more significant effect than performing DA on the decrease in SWDs.

When the microphysics scheme was changed from WSM5 to Morrison, the LWDs increased by +0.37% (+0.56%) in EXP3 (EXP4) compared with EXP1 (EXP2). When DA was performed, the LWDs increased by +0.43% (+0.62%) in EXP2 (EXP4) compared with EXP1 (EXP3). When changing the microphysics scheme from WSM5 to Morrison, the SWD decreased by −5.33% (−6.74%) in EXP3 (EXP4) compared with EXP1 (EXP2). When DA was performed, SWD decreased by −1.87% (−3.33%) in EXP2 (EXP4) compared with that in EXP1 (EXP3). When the microphysics scheme was changed from WSM5 to Morrison or when DA was performed, the LWD increased and the SWD decreased, owing to the increase in cloud liquid water, as shown in §3.1. The effect of cloud ice water on downward radiative flux is much smaller than that of cloud liquid water [22,24]. Using the same experimental framework as this study, Kim and Kim [43] showed that increased LWD and decreased SWD by more simulated cloud liquid water reduced the forecast errors for LWD and SWD.

Therefore, the effects of the microphysics schemes and DA on the LWD and SWD simulations are different, as shown in figure 3*h,i*. Compared with the effect of changing the microphysics scheme from WSM5 to Morrison, the effect of performing DA was greater for the increase in LWD. With respect to the decrease in SWD, the effect of changing the microphysics scheme was greater than that of performing the DA. When Morrison was used instead of WSM5, cloud liquid water increased in the middle and lower atmospheres (figure 10*b*), whereas cloud liquid water increased intensively in the lower atmosphere when DA was performed (figure 10*c*). The variation in SWD is related to the optical thickness of the entire atmospheric layer. Therefore, more cloud liquid water simulated in the entire atmospheric layer using Morrison instead of WSM5 decreased SWD. The variation in LWD is related to the altitude and temperature of the cloud bottom. Therefore, more cloud liquid water simulated in the lower atmosphere by DA increased the LWD.

## 4. Summary and discussion

In the Arctic, clouds affect the radiative flux at the surface and the surface energy budgets; however, the simulation results for Arctic clouds in NWP models are uncertain. These uncertainties are major factors contributing to the discrepancies in the NWP model predictions of the Arctic atmospheric environment. In this study, the effects of the microphysics scheme and DA on the simulation of clouds, hydrometeors and radiative fluxes around Svalbard in the Arctic were investigated.

When the microphysics scheme was changed from WSM5 to Morrison, cloud ice water in the atmosphere decreased by approximately 68%, regardless of the DA. As cloud ice water decreased, water vapour, cloud liquid water, snow and rain increased in the atmosphere, which made the cloud liquid water content similar to CloudSat observations. In contrast, cloud ice water became more similar to CloudSat observations when using WSM5 instead of Morrison in the Arctic. These mixed results for cloud liquid water and ice water contents simulated in the Arctic using Morrison and WSM5 indicate uncertainties associated with Arctic cloud and hydrometeor simulations in NWP models, which requires further case studies using observational and numerical approaches.

When DA was performed, the amount of water vapour increased overall in the atmosphere. The area where water vapour increased by DA was not correlated with the area where the amount and distribution of hydrometeors changed as different microphysics schemes were used. DA modified the amount and distribution of atmospheric water vapour in the initial conditions of the NWP models to be close to the observation data, which means that DA could add more or less water vapour to the model background using observation information. The cloud liquid water was simulated more in the east of Svalbard where the amount of water vapour increased when performing DA. In contrast, when the microphysics scheme was altered, the amount and distribution of water vapour, precipitation and hydrometeors were rearranged in a given physical model environment.

The effects of using Morrison instead of WSM5 or performing DA on cloud liquid water simulation were analysed because cloud liquid water has a greater influence on downward radiative flux than cloud ice water. The cloud liquid water increased in the middle and lower atmospheres when using Morrison instead of WSM5 and increased in the lower atmosphere when DA was performed. In particular, cloud liquid water increased more in the lower atmosphere when DA was performed with Morrison rather than WSM5. As the cloud liquid water increased, the SWD decreased and the LWD increased. Thus, changing the microphysics scheme from WSM5 to Morrison had a greater effect on the decrease in SWD than DA, whereas DA had a slightly greater effect on the increase in LWD than changing the microphysics scheme from WSM5 to Morrison.

Kim and Kim [43] showed that when the microphysics scheme was changed from WSM5 to Morrison and satellite DA was performed, cloud liquid water increased, and therefore forecast errors of both clouds and downward radiative flux were reduced. Thus, based on the results of this study, microphysics schemes could be improved and developed in a way that can maximize their impact on the reduction of forecast errors of downward radiative flux, as well as reduce uncertainties in Arctic cloud simulations. The areas where hydrometeor simulations changed by performing DA were different from the areas where hydrometeor simulations changed as different microphysics schemes were used. Compared with the microphysics scheme of redistributing hydrometeors through physical relationships, DA makes simulation results similar to the real atmospheric environment by assimilating observation data to model simulations. Therefore, the microphysics scheme and DA worked differently, and using them together will be useful for improving hydrometeor simulations in the Arctic.

## Data Availability

The ERA5 reanalysis data are available at [66]. The Prepared Binary Universal Form for the Representation of Meteorological Data is available at [67]. The satellite observations such as Advanced Microwave Sounding Unit-A (AMSU-A) radiance data and Microwave Humidity Sounder (MHS) radiance data are available at [68]. The experimental results are available from the cluster of the National Center for Meteorological Supercomputer of the Korea Meteorological Administration (KMA) (http://super.kma.go.kr).
